# Editorial: Immunological Effects of Nano-Imaging Materials

**DOI:** 10.3389/fimmu.2022.886415

**Published:** 2022-03-24

**Authors:** Yang Li, Pengfei Zhang, Ben Zhong Tang, Diana Boraschi

**Affiliations:** ^1^ Shenzhen Institute of Advanced Technology (SIAT), Chinese Academy of Science (CAS), Shenzhen, China; ^2^ Laboratory of Immunology and Nanomedicine & China-Italy Joint Laboratory of Pharmacobiotechnology for Medical Immunomodulation, Shenzhen Institute of Advanced Technology (SIAT), Chinese Academy of Sciences (CAS), Shenzhen, China; ^3^ Shenzhen Institute of Aggregate Science and Technology, School of Science and Engineering, The Chinese University of Hong Kong, Shenzhen, China; ^4^ Institute of Biochemistry and Cell Biology (IBBC), National Research Council (CNR), Napoli, Italy; ^5^ Stazione Zoologica Anton Dohrn, Napoli, Italy

**Keywords:** nanomaterials, immunity, imaging, inflammation, cancer, diagnosis, immunotherapy

The biomedical application of novel engineered nanomaterials (ENM) has substantially expanded in the last years in many areas, including imaging-based diagnostic approaches and use as imaged-guided carriers for chemotherapeutic drugs and vaccines. In this context, the interaction between nanomaterials for biomedical imaging (nano-imaging materials) and the immune system is a key issue in the safety perspective, as nanomaterial-induced changes in immune reactivity may pose health problems and even participate to disease development. On the other side, such interaction, in particular with cells of the innate immune system, can be exploited for the targeted activation of beneficial immune functions, thereby opening the way to new immunotherapeutic strategies targeting innate immunity.

The Research Topic “*Immunological Effects of Nano-Imaging Materials*” aims at providing new information and perspectives of the immune-related effects of nano-imaging materials, used or developed for diagnostic procedures, which allow us to predict a much wider exploitation that encompasses immunotherapeutic and immunopreventive purposes. The synergy between three different research areas and related Frontiers journals (Frontiers in Immunology, Frontiers in Bioengineering and Biotechnology, Frontiers in Molecular Biosciences) provides a wide interdisciplinary view of different technological and conceptual strategies and underlines the need for a comprehensive approach for successfully addressing and exploiting the capacity of nanomaterials to interact with the immune system (**Figure 1**).

**Figure 1:**
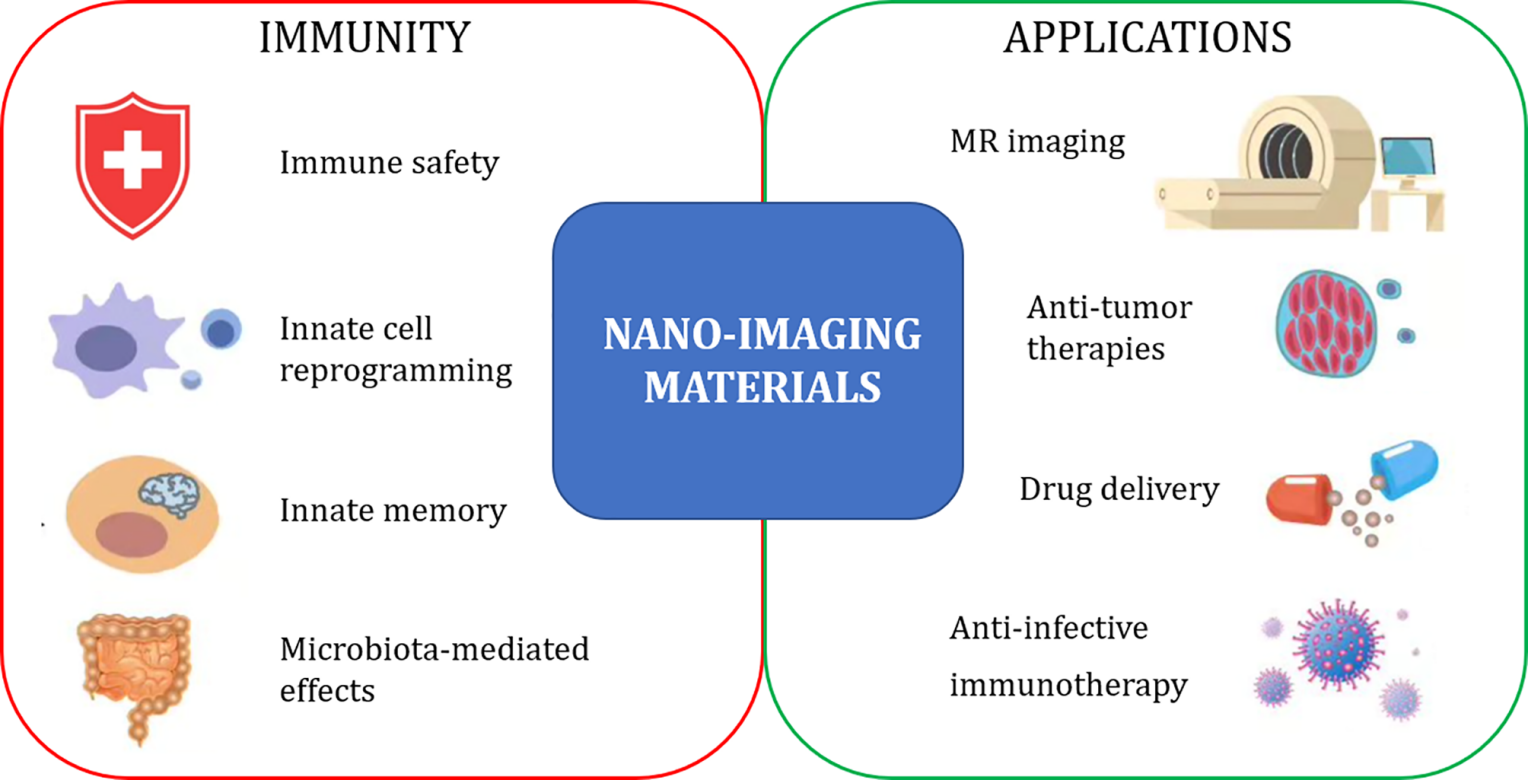
Immunological effects and biomedical application of nano-imaging materials.

Safety, in particular immunosafety, is the primary issue for any new nanomaterial to be used in nanomedicine. Our immune system is devoted to surveillance and protection of the body integrity from endogenous and exogenous agents, such as senescent/anomalous cells, pathogens and particles. In this scenario, ENMs used in medical applications may trigger a defensive immune reaction, which may cause immune-related adverse effects and/or the failure of medical treatment. Thus, the immunological effects and immunosafety of the nano-imaging materials should be carefully evaluated before their clinical application, so as to allow for their optimal application in the medical practice through a safer-by-design ENM optimisation.

We have examined here the immune-related toxicity and effects of some new nanomaterials with unique physico-chemical and optical characteristics, in particular Aggregation-Induced Emission (AIE) imaging materials, 2D nano-imaging materials, quantum dots, and perovskite nanomaterials (Wu et al., Da Silva et al *.*, Geppert and Himly, and Wei et al.).

Numerous organic and nanomaterial-based fluorophores possess excellent fluorescent properties when dispersed in solution. However, aggregation of these materials is likely to result in fluorescent quenching due to the ACQ effect, which is largely responsible for nonradiative energy transfer induced by strong π-π stack. Since the discovery of AIE effects in 2001, great efforts have been made to exploit AIE-active materials in medical applications for diagnosis and treatment, as summarized by Wu et al.

The group of Diego Martinez reviewed the most recent advances relative to the immunological effects of the two dimensional (2D) ENM applied to bio-imaging, and highlighted to use nanoinformatics approaches/computational modelling for nano-immunosafety evaluation, aiming at providing key information towards immunologically safer design of 2D-ENM (Da Silva et al *.)*.

Geppert and Himly discussed the immunological effects induced by iron oxide nanoparticles (IONP) used as MRI contrast agent, and described how the physico-chemical properties of IONP are crucial in determining their biological and immunological effects. Notably, they stressed an important issue in the assessment of nano-immunosafety, *i.e.*, the undetected presence of endotoxin (lipopolysaccharide, LPS), a ubiquitous bacterial molecule with strong inflammatory/toxic effects (1). Thus, the association of immunoactive agents such as LPS to the surface of nanoimaging materials (usually occurring during nanomaterial synthesis or storage) must be assessed and prevented, in order to improve the material safety and correctly assess its biological effects. In this respect, Anna Chiara De Luca’s group has developed a highly sensitive surface-enhanced Raman spectroscopy (SERS) method for accurate endotoxin detection in nano-imaging gold ENM, which overcomes the many issues of optical interference experienced when the current endotoxin detection assays are applied to ENM (Verde et al.) Such evaluation is fundamental for toxicological evaluation of nanomaterials, in order to discriminate between nano-dependent and contaminant-dependent toxicity. On the other hand, the interaction of nanomaterials with bacteria can also have very promising health-improving applications, as in the case of interaction with gut microbiota, which can change the microbial composition in the gut and modulate in a positive fashion the microbiota-dependent immune functions, as pointed out by Liang Li’s group (Tang et al.).

The group of Shanze Chen reviewed the biomedical application and immunological responses of a traditional nano-imaging material, quantum dots (QD), with focus on the respiratory apparatus (Ren et al.). They highlighted the adverse effects and immunosafety concerns caused by QD, which are mainly related to oxidative stress caused by the release of toxic metal ions and reactive surface charge. Reactive oxygen species generated by ENM could be considered as a common reason for ENM-induced cyto/immuno-toxicity, as they seem to also account for the immuno-toxic effects of Perovskite NM observed by the Liming Wang’s group (Wei et al.). However, there are reports on many other potentially toxic effects of medical ENM, relevant to immunotoxicity, for instance the specific molecular interference with cell duplication (2), an effect that may hamper lymphocyte proliferation during adaptive immune responses. This asks for a deeper molecular analysis of nano-immune interactions, in order to clearly define the possible immunotoxicity risks on different types of immune cells/organs/responses.

Thus, by examining the effects of nanoimaging materials on immunity, we can foresee the possibility of activating or modulating some specific immune-related functions for bioimaging-guided therapeutic purposes.

This is the case of nano-based strategies for modulating innate memory, reported by Paola Italiani’s group, strategies that would make possible to change/improve the capacity of innate immune cells to mount a defensive response to infections or diseases (Della Camera et al.). The effect on innate immunity has the advantage of being largely non-specific, meaning that a positive nano-effect would apply to a range of different diseases, but it seems to depend on the individual subject (*i.e.*, each human being has her/his own individual way to generate innate memory in response to nanoparticles), calling for a personalized profiling and nano-application (Italiani et al.).

The capacity of EMNs to induce and direct innate immune cells activation (*e.g.*, anti-inflammatory macrophage polarization) can be harnessed for improving tissue replacement integration. The use of nano-patterned titania described by Ting Zhang and colleagues can induce macrophage anti-inflammatory activation with the release of exosomes able to facilitate bone cell differentiation and bone apposition (Zhang et al.), thereby opening the way to the design of “intelligent” biomaterials for tissue replacement that facilitate transplant take and integration.

In the case of immunotherapeutic approaches to cancer, IONP can be of particular interest for their ability to drive the anti-tumor capacity of tumor-associated macrophages, in addition to direct cytotoxic effects on tumor cells, as reported by Yang Li’s group (Song et al.). Other novel “intelligent” photoreactive bimetallic nanoparticles can be designed, able to induce tumor cell death by inducing a strong oxidative burst and localized hyperthermia within the hypoxic tumor microenvironment (Li et al.). Particles can be designed with different characteristics for adding functional properties, as shown in the exosome-camouflaged particles that can act as chemotherapeutic drug carriers in addition to their photothermal therapeutic properties (Tian et al.). Other particles, such as the self-assembling amphiphilic nanodots reported by Yang et al., can unite their ability to deliver chemotherapeutic agents with the AIE-based capacity for cancer imaging and accurate targeting.

Lastly, nano-probes sensitive to changes in pH and/or oxidation can be used for imaging of pathological innate immune reactions, such as trauma-induced neuroinflammation, which can be identified by detection of hypoxia-induced pH decrease and the sustained production of reactive oxygen species, as described by Lu et al., while AIE luminogen-based nanodots are being applied to improving the specificity and sensitivity of detection assays for SARS-CoV-2, because of their high stability and lack of non-specific luminescence, as reported by Liu et al.

While our current knowledge on nano-immune interactions is fostering the development of many tools and strategies for using nano-imaging materials for the targeted therapeutic activation of immunity, it is also clear that much basic information is still missing, which would need a stricter collaboration between immunologists, nanotechnologists, pharmacologists, toxicologists and clinicians in order to tackle and overcome current gaps and weaknesses. This Research Topic aims to be a first practical step in this direction.

## Author Contributions

All authors contributed to writing and revised the manuscript.

## Funding

YL is supported by National Natural Science Foundation of China (32171390), International Partnership Program (IPP) of CAS (172644KYSB20210011), National Key R&D Program of China (2021YFE0113000), Shenzhen Science and Technology Program (GJHZ20190821155803877), CAS President’s International Fellowship Initiative (PIFI, 2021PB0060, 2022VBA0008). PZ is supported by National Key R&D Program of China (2019YFE0198700), Shenzhen Science and Technology Program (JCYJ20210324120011030). DB is supported by the EU H2020 project PANDORA (GA 671881) and by CAS-PIFI (2020VBA0028).

## Conflict of Interest

The authors declare that the research was conducted in the absence of any commercial or financial relationships that could be construed as a potential conflict of interest.

## Publisher’s Note

All claims expressed in this article are solely those of the authors and do not necessarily represent those of their affiliated organizations, or those of the publisher, the editors and the reviewers. Any product that may be evaluated in this article, or claim that may be made by its manufacturer, is not guaranteed or endorsed by the publisher.
